# CoMR: an integrative scoring pipeline for comprehensive mitochondrial proteome reconstruction across eukaryotes

**DOI:** 10.1093/bib/bbag474

**Published:** 2026-09-07

**Authors:** Julie Boisard, Shelby K Williams, Andrew J Roger, Courtney W Stairs

**Affiliations:** Department of Cell and Molecular Biology, Uppsala University, Husargatan 3, 752 37 Uppsala, Sweden; Department of Biology, Lund University, Sölvegatan 35, 223 62 Lund, Sweden; Institute for Comparative Genomics, Dalhousie University, 5850 College Street, Halifax, Nova Scotia B3H 4R2, Canada; Department of Biochemistry and Molecular Biology, Dalhousie University, Sir Charles Tupper Medical Building, 5850 College Street, Halifax, Nova Scotia B3H 4R2, Canada; Institute for Comparative Genomics, Dalhousie University, 5850 College Street, Halifax, Nova Scotia B3H 4R2, Canada; Department of Biochemistry and Molecular Biology, Dalhousie University, Sir Charles Tupper Medical Building, 5850 College Street, Halifax, Nova Scotia B3H 4R2, Canada; Department of Cell and Molecular Biology, Uppsala University, Husargatan 3, 752 37 Uppsala, Sweden; Department of Biology, Lund University, Sölvegatan 35, 223 62 Lund, Sweden; Science for Life Laboratory (SciLifeLab), Uppsala University, Husargatan 3, Uppsala 752 37, Sweden

**Keywords:** mitochondrial proteome reconstruction, mitochondrion-related organelles, organelle evolution, mitochondrial targeting signals prediction, integrative evidence scoring, reproducible bioinformatics workflow

## Abstract

Mitochondrial proteome reconstruction from eukaryotic sequence data typically relies on prediction of mitochondrial targeting signals (MTSs). However, MTS predictors are primarily trained on model organisms and may perform poorly in phylogenetically divergent lineages or in organisms with atypical or reduced targeting sequences. Accurate reconstruction therefore requires integration of complementary sources of evidence beyond targeting prediction alone. We developed Comprehensive Mitochondrial Reconstructor (CoMR), an integrative workflow that combines targeting prediction, curated homology searches, large-scale similarity searches, and automated phylogenetic analysis within a unified scoring framework. Benchmarking on the model yeast *Saccharomyces cerevisiae* yielded strong discriminatory performance [receiver operating characteristic (ROC)-area under the curve (AUC) = 0.92], exceeding standalone prediction with TargetP2, a predictor of N-terminal targeting peptides (ROC-AUC = 0.72). In the divergent anaerobic protist *Paratrimastix pyriformis*, CoMR maintained robust performance (ROC-AUC = 0.86) validated with an experimental proteome despite extreme class imbalance, achieving a precision-recall AUC of 0.183 (~78-fold enrichment over random expectation and ~10-fold improvement over TargetP2). Ablation analyses demonstrate that predictive performance is robust to individual evidence-layer removal, while overlap analyses showed that homology-based searches recovered candidates missed by targeting predictors, particularly in *P. pyriformis*. Overall, CoMR improves mitochondrial proteome reconstruction over targeting prediction alone and provides a reproducible workflow for predicting mitochondrial and mitochondrion-related organelle protein repertoires across eukaryotes to aid investigations of organelle evolution and proteome reduction.

## Introduction

Mitochondria are central to eukaryotic cellular metabolism, participating in adenosine triphosphate (ATP) production through oxidative phosphorylation as well as amino acid metabolism, iron–sulfur cluster biogenesis, and organellar genome maintenance [1–3]. In well-studied aerobic organisms such as humans and yeast, mitochondrial proteomes have been extensively characterized, providing detailed insight into organellar composition and function [4, 5]. However, accurate reconstruction of mitochondrial proteomes remains challenging in many eukaryotic lineages, particularly those that are phylogenetically divergent or adapted to anaerobic environments.

Anaerobic protists represent some of the most understudied yet phylogenetically diverse branches of the eukaryotic tree of life. Many possess mitochondrion-related organelles (MROs), which are highly reduced versions of canonical mitochondria [6, 7]. MROs vary in structure and metabolic capacity across lineages, often exhibiting lineage-specific adaptations and reductions [8]. Studying these organelles provides insight into mitochondrial evolution and organelle plasticity, but reconstructing their proteomes is computationally and experimentally challenging.

*In silico* prediction of mitochondrial localization typically relies on detection of N-terminal mitochondrial targeting signals (MTSs). Most MTS predictors are trained on model organisms and assume canonical targeting sequence properties. In divergent lineages such as metamonads, MTSs can be highly atypical or absent, and protein import may rely more heavily on internal targeting elements [9–11]. Consequently, targeting-based approaches alone may underestimate mitochondrial proteome size or misclassify divergent proteins. Homology-based searches can complement targeting prediction, but database composition and phylogenetic representation strongly influence performance [12–14]. A unified framework that integrates multiple independent sources of evidence for mitochondrial localization is therefore needed.

Here, we present Comprehensive Mitochondrial Reconstructor (CoMR), an integrative workflow for mitochondrial and MRO proteome reconstruction. Compared with standalone mitochondrial targeting predictors or homology-based annotation strategies, CoMR is designed as a reproducible and configurable framework that integrates multiple sources of evidence. CoMR combines targeting prediction, curated and large-scale homology searches, profile-based hidden Markov model (HMM) detection, and automated phylogenetic analysis within a unified scoring framework. CoMR differs from standalone predictors by combining complementary sources of evidence into a configurable score and by returning ranked candidate lists with the supporting evidence retained for each protein, rather than a single binary prediction. Because each candidate remains linked to its targeting, homology, HMM, and phylogenetic evidence, users can inspect why a protein was prioritized. We benchmark CoMR on experimental datasets from both a model organism (*Saccharomyces cerevisiae*) and a divergent anaerobic protist (*Paratrimastix pyriformis*), demonstrating improved predictive performance over standalone targeting prediction and robust behavior under extreme class imbalance.

## Materials and methods

### Overview of the CoMR workflow

**Figure 1:**
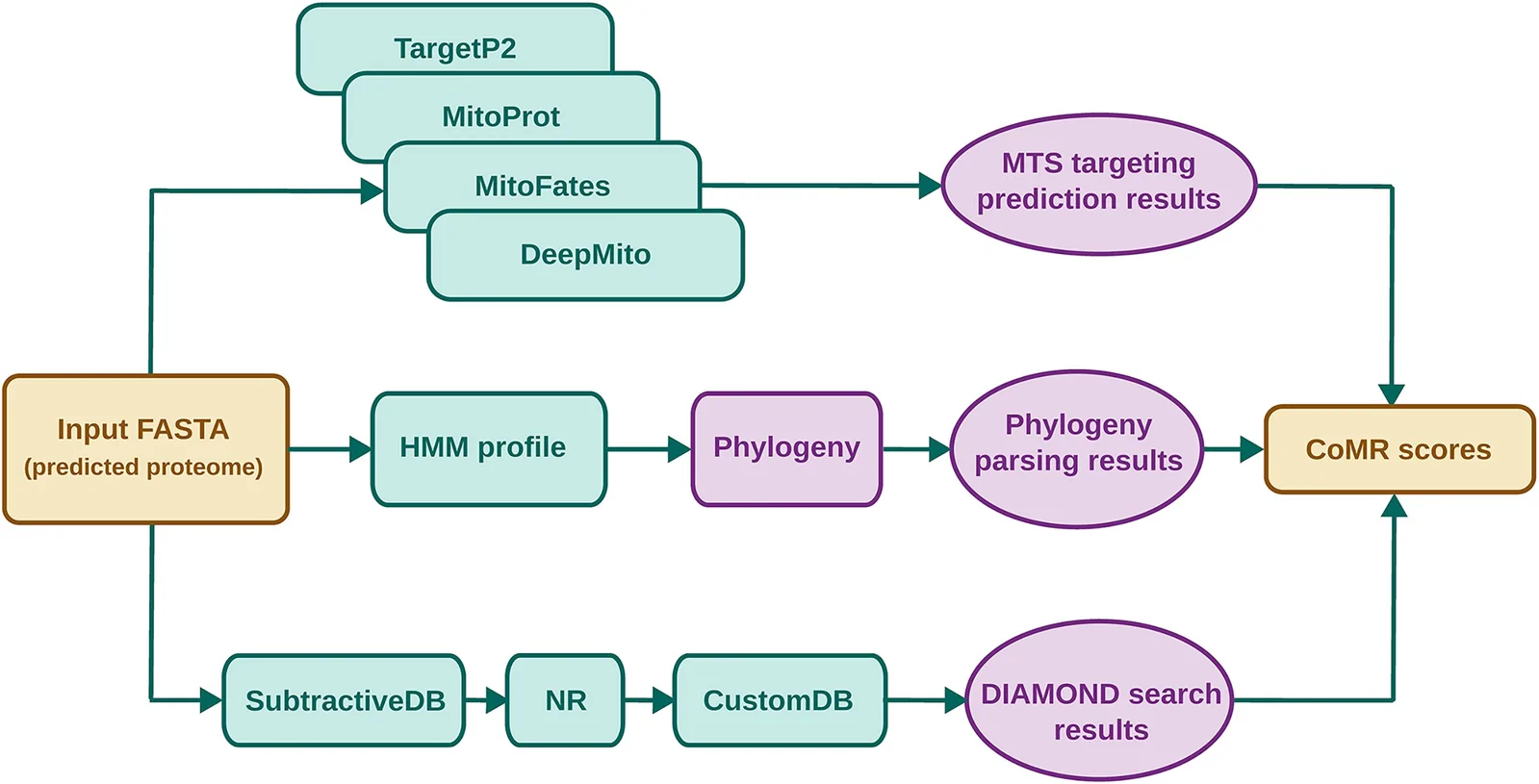
Overview of the CoMR pipeline; CoMR integrates targeting signal prediction, homology searches, and phylogenetic analysis to reconstruct a putative mitochondrial or MRO proteome from a predicted proteome (input proteome; three complementary evidence streams are computed in parallel; MTS prediction branch: TargetP2, MitoProt II, MitoFates, and DeepMito predict MTSs; their outputs are aggregated as MTS targeting prediction results; phylogenetic branch: HMM profile searches identify candidate protein families, followed by phylogenetic reconstruction and automated tree parsing to classify sequences as mitochondrial or non-mitochondrial; sequence similarity branch: Subtractive Mitochondrial Database (SubstractiveDB, SMD), NR, and optional user-defined database for lineage-specific investigations (CustomDB); DIAMOND searches provide homology-based evidence, summarized as DIAMOND search results; all intermediate evidence layers are integrated by a user-defined scoring scheme to produce the final CoMR scores, which summarize mitochondrial support across all evidence categories.

CoMR is a modular Snakemake-based pipeline designed to identify and score mitochondrial and MRO proteins from predicted eukaryotic proteomes. The workflow integrates targeting signal predictors, profile HMMs, homology searches, and phylogenetic validation into a unified evidence framework that produces ranked candidate lists suitable for expert curation (Fig. 1).

### Input data and preprocessing

CoMR accepts one or more predicted proteomes in FASTA format, a text-based format for representing amino acid sequences. The input protein sequences can be derived from gene models, transcriptomes, or proteomic analysis. Each input FASTA undergoes normalization, during which sequence headers are replaced by unique identifiers to ensure compatibility with downstream tools and to enable reproducible joins across evidence sources. A mapping table linking original headers to internal identifiers is retained. A filtered FASTA containing only methionine-initiated sequences is generated for N-terminal targeting prediction steps, while the full indexed FASTA is preserved for homology-based analyses.

### Targeting and localization prediction

Four independent predictors are applied to each normalized proteome to infer MTSs and subcellular localization. TargetP 2.0 [15], a predictor of N-terminal targeting peptides and protein subcellular localization, is used to predict mitochondrial targeting peptides and cleavage sites, while MitoFates [16], a predictor of cleavable N-terminal mitochondrial targeting signals and cleavage sites, and Mitoprot II [17], a predictor of mitochondrial targeting sequences, provide complementary estimates of presequence presence and cleavage positions. DeepMito [18], a deep-learning method for predicting protein sub-mitochondrial localization, is run using a bundled Universal Protein resource (UniPro) [19] reference FASTA to infer localization probabilities. Raw outputs from each predictor are retained, and parsed summary tables are generated to standardize scores and categorical calls across tools.

### Homology and profile-based searches

DIAMOND [20] protein-protein Basic Local Alignment Search Tool (BLASTP) searches are executed against multiple protein databases, including the CoMR Subtractive Mitochondrial Database (SMD). The SMD is derived from the proteome of six organisms (*Andalucia godoyi* [21], *Tetrahymena thermophila* [22, 23], *Arabidopsis thaliana* [24], *Homo sapiens* [25], *Acanthamoeba castellanii* [26, 27], and *S. cerevisiae* [28]) divided into two FASTA files with one file containing the sequences of curated mitochondrial proteins and the other file containing sequences of non-mitochondrial proteins. These six organisms were selected as representatives of diverse major lineages, including opisthokonts, archaeplastids, amoebozoans, alveolates, and jakobids, while relying on species whose mitochondrial proteomes have been experimentally characterized or whose data have been carefully validated. This approach allows the SMD to combine broad phylogenetic coverage with validated mitochondrial and non-mitochondrial reference sets for a subtractive assessment of homology. To capture conserved mitochondrial protein families, CoMR performs profile hidden Markov models (HMM) searches with the biosequence analysis software suite HMMER [29] (hmmscan), using a curated CoMR mitochondrial profile HMM database derived from the SMD using default settings. Significant hits are parsed and used to nominate sequences for downstream alignment and phylogenetic analysis. Input sequences can optionally be used as queries against the National Center for Biotechnology Information (NCBI) non-redundant (NR) database: when NR searches are enabled, taxonomy annotation is reconstructed using NCBI taxonomy dump files [30]. An optional user-defined protein sequence database (CustomDB) search can be activated to use the input proteomes as queries against a user-provided DIAMOND database for lineage-specific analyses. The construction and usage of the SMD are detailed in the Supplementary Data (Supplementary Methods—CoMR databases), along with a description of its usage in CoMR’s profile HMM database, phylogenetic analysis, and tree parsing function. All CoMR databases are available on the CoMR Figshare repository (https://doi.org/10.17044/scilifelab.31361839).

### Alignment and phylogenetic inference

Sequences with significant HMM hits are incorporated into reference multiple sequence alignments using Multiple Alignment using Fast Fourier Transform (MAFFT 7) [31], followed by automated trimming with trimAl to remove poorly aligned regions. For each trimmed alignment, phylogenetic trees are inferred using IQ-TREE 2 [32], a software package for maximum-likelihood phylogenetic inference, under Le and Gascuel (LG) amino acid substitution model with gamma-distributed rate heterogeneity (+G) with fast heuristics enabled (-fast). Trees are parsed with ete3 [33]. Mitochondrial and non-mitochondrial localizations are treated as discrete states and assigned to the reference leaves in the tree. Internal-node states are then reconstructed by Fitch parsimony, and each query sequence is assigned the state inferred for the attachment point at which it joins the rest of the tree, with unresolved mitochondrial/other ambiguity retained as undefined [34].

### Evidence integration and scoring

All parsed evidence layers, including targeting predictions, HMMscan hits, blast homology scores and taxonomy, and phylogenetic classifications, are merged into a single per-sequence table. CoMR applies a configurable scoring rubric defined in external scorecard files, allowing flexible weighting of individual evidence sources.

CoMR integrates heterogeneous evidence sources at the level of individual protein sequences using a rule-based scoring framework. Each layer of evidence provides a clue as to the presence or absence of support for mitochondrial localization. Under the default scoring scheme, each qualifying evidence source contributes equally to a cumulative CoMR score, which is reported alongside detailed per-component annotations to ensure interpretability and traceability. The default equal-weight scoring system was chosen to prioritize transparency and interpretability rather than dataset-specific optimization. Because CoMR is intended for use across diverse eukaryotic lineages with variable database representation and targeting-signal properties, equal weighting provides a conservative default, while the configurable scorecard allows users to adjust evidence weights according to lineage-specific knowledge, database availability, or research question.

A sequence receives one point for each mitochondrial targeting predictor reporting a positive prediction (TargetP2, MitoProt, or MitoFates), one point for curated homology support based on matches to multiple mitochondrial datasets in the SMD, one point for phylogenetic placement within a mitochondrial clade, and one point for large-scale homology support from the NCBI NR database when hits are annotated with mitochondrion-related keywords (i.e. mitochondrion, mitochondria, mitochondrial, hydrogenosome, hydrogenosomal, mitosome, and mitosomal). DeepMito predictions are not included in the composite score, as this tool assumes mitochondrial localization and instead provides submitochondrial localization information. DeepMito outputs are reported alongside the composite score to facilitate downstream interpretation. The default equal-scoring scheme assigns equal weight to all contributing evidence layers, resulting in a composite score ranging from 0 to 6. Alternative scoring schemes can be defined by modifying the scorecard file provided with CoMR. The detailed binarization rules and scoring logic are described in Supplementary Data (Supplementary Methods—CoMR evidence integration and scoring).

### Workflow execution and containerization

CoMR is distributed as a fully containerized workflow to ensure reproducibility across computing environments. CoMR’s Docker [35] image and Singularity/Apptainer [36] image can be retrieved from the CoMR GitHub repository (https://github.com/theLabUpstairs/CoMR). Licensed software (TargetP2) and large external databases are mounted at runtime rather than bundled in the image. The workflow is executed via Snakemake within the container, with user-configurable resource limits for parallelization and memory-intensive steps such as DIAMOND searches.

### Software environment

The runtime environment is defined via a Conda specification in the container and includes Python 3.11, Snakemake [37], HMMER, DIAMOND, MAFFT, IQ-TREE 2, Biopython [38], pandas, and supporting libraries required for parsing, scoring, and phylogenetic analysis.

### CoMR performance evaluation

Benchmarking was performed on one model organism (*S. cerevisiae*) and one non-model organism (*P. pyriformis*). *P. pyriformis* was chosen over metamonads such as *Giardia intestinalis* and *Trichomonas vaginalis* because it represents a more divergent and less sampled lineage while still providing an experimentally supported MRO proteome and a whole-cell proteome for benchmarking. Its small curated MRO proteome also serves as a test case with a significant imbalance between classes, allowing the evaluation of CoMR under conditions that more closely resemble mitochondrial proteome reconstruction in poorly represented non-model eukaryotes.

To prevent circularity, benchmarking-specific versions of all internal reference databases were generated by excluding sequences from the evaluated species prior to homology searches. In addition, DIAMOND searches against the NCBI NR database were parsed using a taxonomically controlled exclusion strategy, in which hits assigned to the evaluated species or higher taxonomic ranks were removed prior to evidence integration. Performance was assessed using threshold-based metrics, receiver operating characteristic (ROC)-area under the curve (AUC) and precision-recall (PR) analyses across CoMR score thresholds (0–6) and compared to individual mitochondrial targeting predictors using their recommended cutoffs. To quantify the contribution of individual evidence layers, ablation effects were reported as the change in ROC-AUC relative to the default equal-weight CoMR score. To assess redundancy between evidence classes, we compared MTS-based and homology-based support among reference positives. MTS support was defined as a positive prediction by at least one targeting predictor: TargetP2, MitoProt, or MitoFates. Homology support was defined as positive support from either SMD or NR. Proteins were classified as MTS-only, homology-only, supported by both evidence classes, or supported by neither. Pairwise overlap between individual evidence layers was additionally quantified using the Jaccard index and reported in the Supplementary Table S4. Because CoMR produces ranked candidate lists, we also evaluated practical prioritization performance by measuring the enrichment of reference mitochondrial/MRO proteins among the top 10, 25, 50, 100, 250, 500, and 1000 candidates ranked by CoMR score. For each Top-*N* subset, we calculated true positives (TPs), precision, recall, and fold enrichment over random expectation; ties were resolved deterministically by protein identifier.

Detailed benchmarking is described in Supplementary Data (Supplementary Methods—CoMR performance evaluation) and available on the CoMR Figshare repository (CoMR_DB_hmm_benchmarking, https://doi.org/10.17044/scilifelab.31361839).

## Results

### Overview of CoMR outputs and composite scoring

For each input proteome, CoMR produces a unified evidence table in which all targeting predictions, homology-based signals, and phylogenetic classifications are integrated at the level of individual protein sequences. Each sequence is assigned a composite CoMR score summarizing the number of independent lines of evidence supporting mitochondrial localization. The output table reports, for each sequence, the composite CoMR score together with the individual contributions of targeting predictors, homology searches, phylogenetic classification, and associated annotation fields. Under the default “equal” scoring scheme, the CoMR score ranges from 0 to 6, with higher values indicating increasing support from multiple evidence layers, including mitochondrial targeting predictions, curated homology matches, large-scale homology searches, and phylogenetic placement. DeepMito predictions are reported alongside the score to provide submitochondrial localization information but are not included in the composite score. The resulting score provides a continuous ranking of candidate proteins rather than a binary classification, allowing users to apply dataset-specific thresholds or to prioritize high-scoring sequences for downstream analyses and mitochondrial proteome reconstruction. A schematic overview of the CoMR workflow is shown in Fig. 1.

### Benchmarking strategy and evaluation framework

To evaluate the performance of CoMR, we benchmarked its ability to predict mitochondrial localization using both a well-annotated model organism (*S. cerevisiae*) and a non-model metamonad (*P. pyriformis*). This design allowed us to assess CoMR performance under conditions of extensive reference data availability as well as in a lineage with highly reduced and divergent mitochondrial-related organelles. To prevent circularity during benchmarking on *S. cerevisiae*, all internal reference databases used by CoMR were reconstructed after excluding *S. cerevisiae* sequences, as described in the Supplementary Data (Supplementary Methods—CoMR performance evaluation). In addition, homology evidence derived from the NCBI NR database was controlled by taxonomic exclusion of hits assigned to *S. cerevisiae* or its close relatives. Benchmarking was performed under multiple exclusion stringencies (species-, genus-, and order-level), with stricter conditions reported in the Supplementary Data (Supplementary Methods—CoMR performance evaluation, Supplementary Table S1, and Supplementary Fig. S1).

For *P. pyriformis*, benchmarking was performed using both the whole-cell proteome and a curated MRO proteome [39], with taxonomic exclusion of its NCBI taxonomic identifier from NR-derived evidence. CoMR performance was evaluated using the default equal-weight scoring scheme, in which all six evidence layers contribute equally to the composite score (range 0–6). For each dataset, the true positive rate (TPR) and false positive rate (FPR) were calculated across all score thresholds and used to construct ROC curves and corresponding AUC (ROC-AUC) values. CoMR performance was compared to the baseline performance of TargetP2 using identical reference sets. Because mitochondrial proteins represent a small fraction of the *P. pyriformis* proteome, PR curves and area under the PR curve values were additionally computed for this dataset.

### CoMR performance on a model organism, *Saccharomyces cerevisiae*

**Figure 2:**
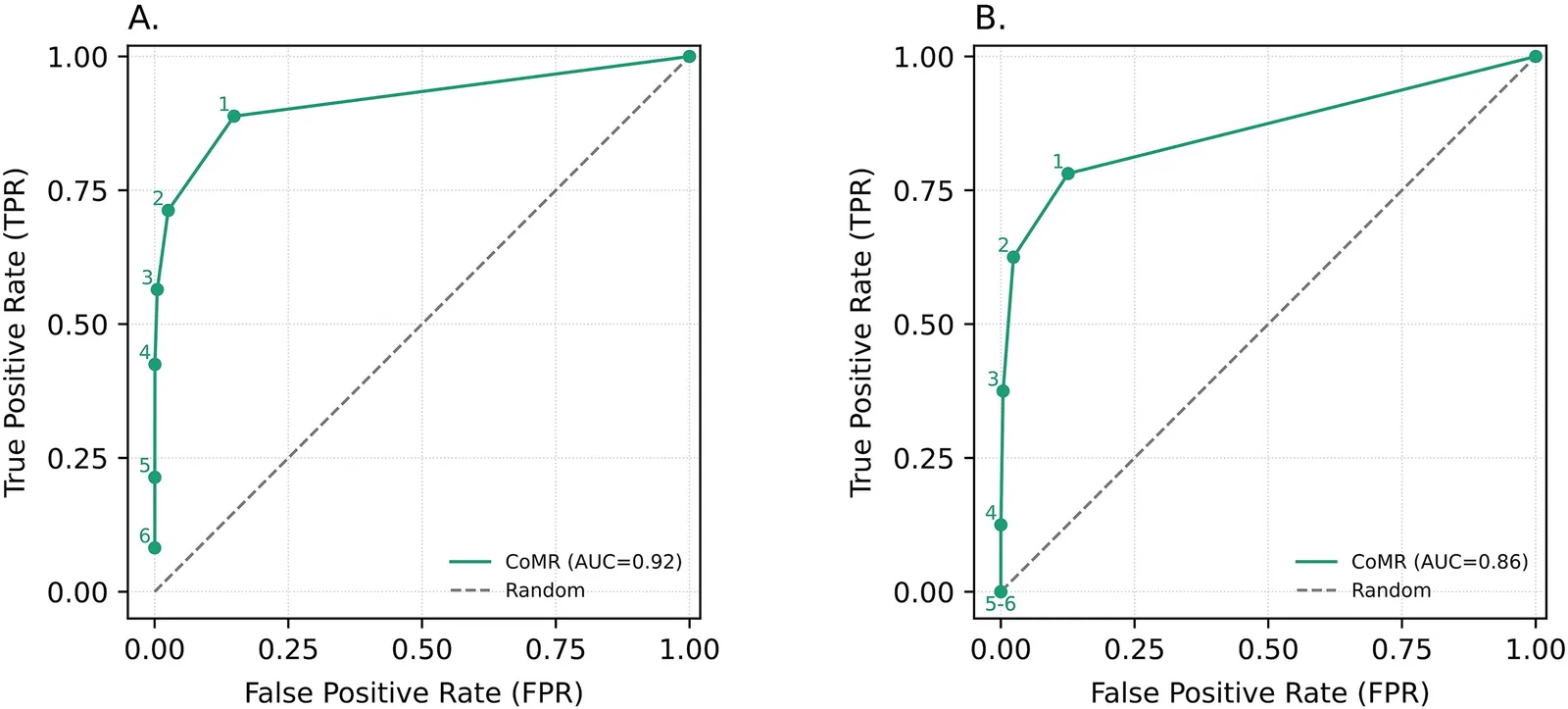
The threshold-resolved ROC curves for *S. cerevisiae* proteome (AUC = 0.92) and *P. pyriformis* proteome (AUC = 0.86) indicate strong performance, with CoMR maintaining high sensitivity at low FPRs across score cutoffs; (a) *S. cerevisiae* proteome; (b) *P. pyriformis* proteome; the ROC curves were computed using the equal scoring scheme, with possible scores ranging between 0 and 6 (noted next to data points); note that scores 5 and 6 for *P. pyriformis* share the same data point; the dashed line represents the ROC curve for a classifier that randomly assigns positive and negative classes.

CoMR was applied to the *S. cerevisiae* proteome to evaluate its ability to distinguish mitochondrial from non-mitochondrial proteins. CoMR scores were compared to the curated reference proteome [28], and TPR and FPR values were calculated across all possible score thresholds (0–6). These values were used to construct a ROC curve for the default equal-weight scoring scheme (Fig. 2a). The resulting area under the ROC curve (ROC-AUC) was 0.92, indicating strong discriminatory performance of CoMR on the *S. cerevisiae* proteome.

**Figure 3:**
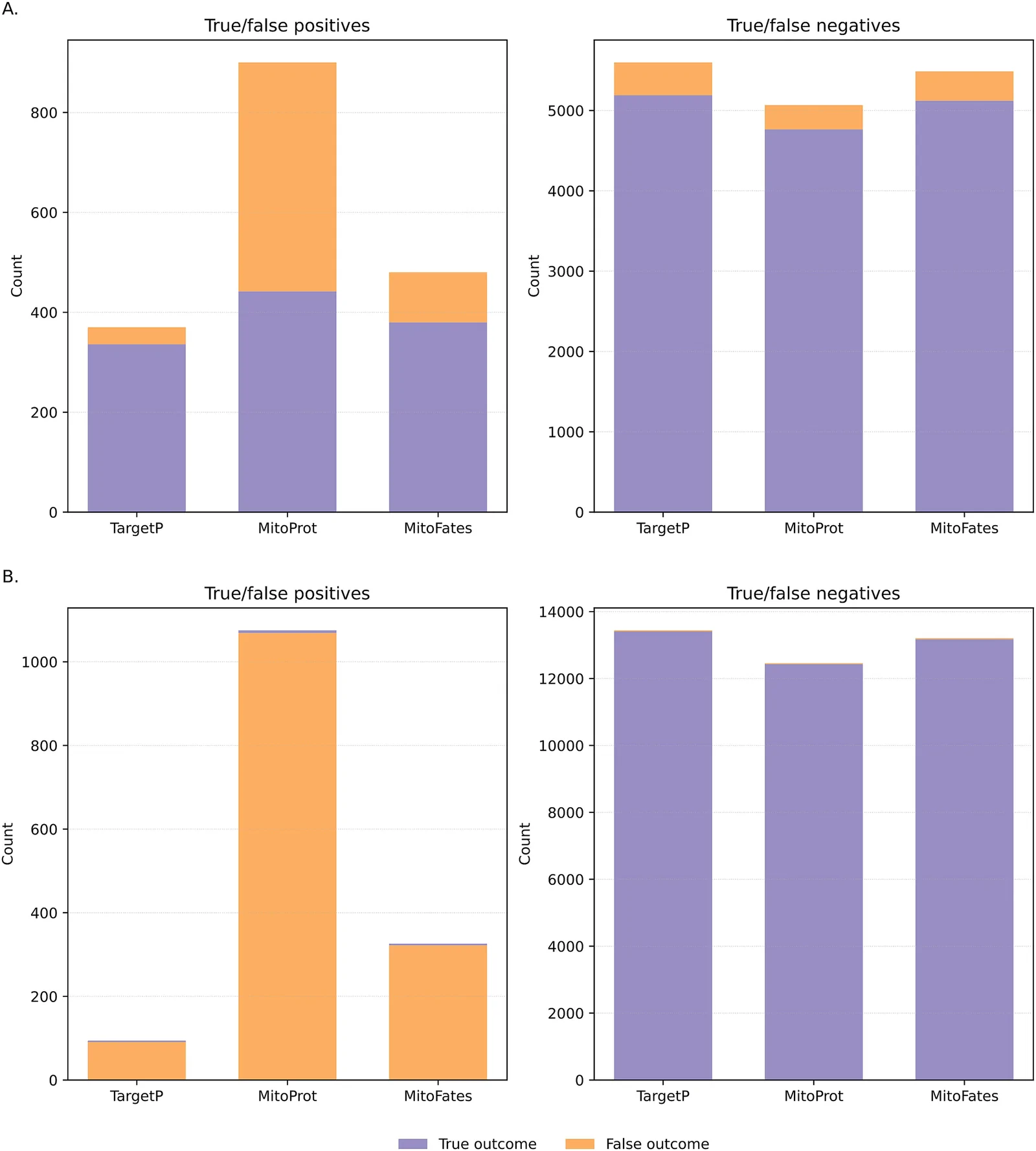
MTS predictor performance in the *S. cerevisiae* proteome and *P. pyriformis* proteome; (a) *S. cerevisiae* proteome; (b) *P. pyriformis* proteome; stacked bar plots compare confusion counts for TargetP2, MitoProt, and MitoFates at their CoMR recommended thresholds.

**Figure 4:**
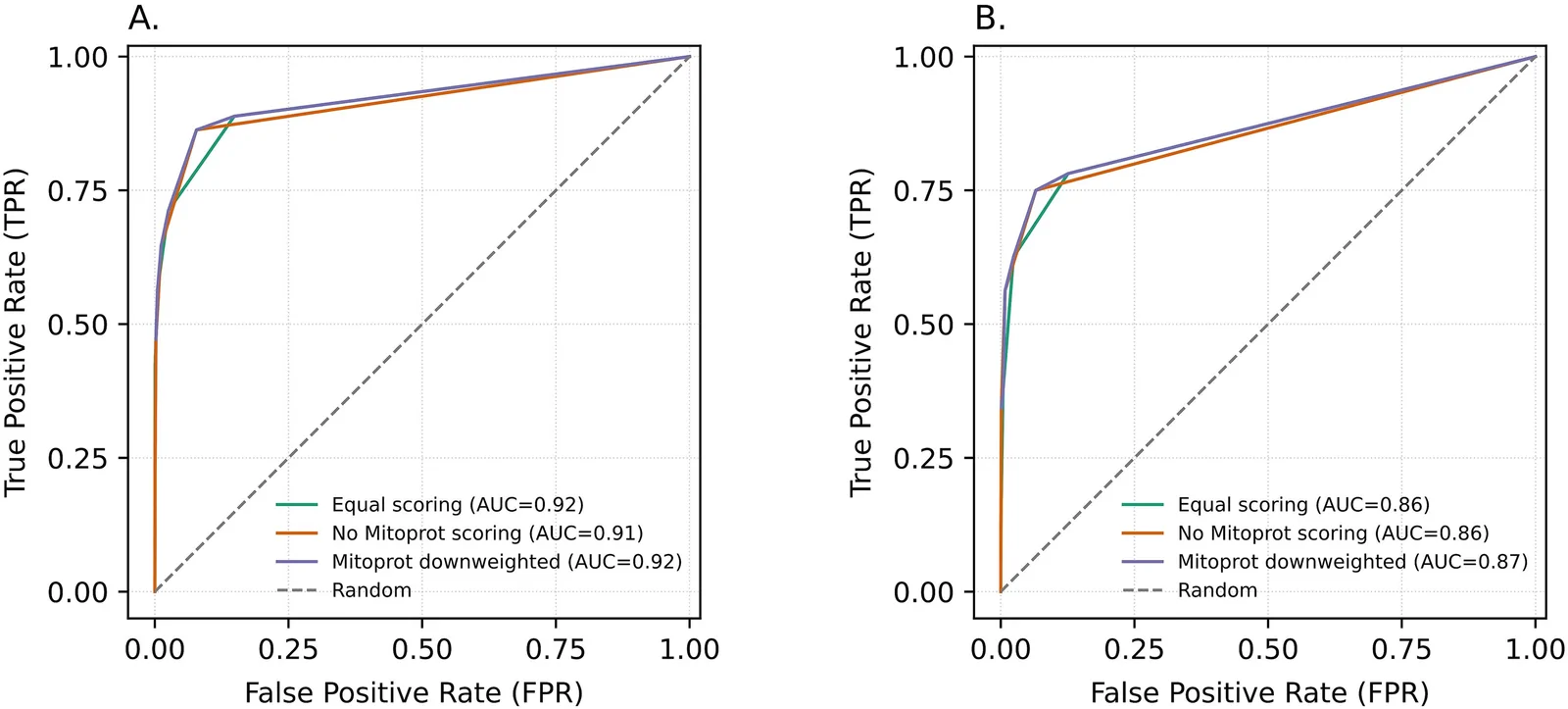
MitoProt weighting slightly influences CoMR ROC performance in the *S. cerevisiae* proteome and *P. pyriformis* proteome; (a) *S. cerevisiae* proteome; (b) *P. pyriformis* proteome; ROC curves were computed under three MitoProt-related scoring schemes: equal scoring, no MitoProt scoring, and MitoProt down-weighted; each curve is annotated with its AUC; the dashed line indicates a random classifier.

We first examined the baseline performance of the three MTS predictors on the *S. cerevisiae* proteome by comparing TP, false positive (FP), true negative, and false negative (FN) counts for each predictor. All three predictors showed strong ability to correctly identify non-mitochondrial proteins, with relatively low FN rates (Fig. 3a). However, their precision differed, with MitoProt producing a higher number of FPs compared to TargetP2 and MitoFates. To assess the impact of this behavior on CoMR performance, we evaluated alternative scoring schemes in which MitoProt was either excluded entirely or down-weighed by reducing its contribution to the overall score (Fig. 4a). These modified schemes allowed us to determine whether MitoProt-associated FP disproportionately influenced CoMR’s predictive accuracy.

We then recalculated TPR and FPR values under these modified scoring schemes and reconstructed the corresponding ROC curves and ROC-AUC values (Fig. 4a and Table 1). Down-weighting MitoProt (0.5 instead of 1 point) slightly improved ROC-AUC, whereas complete removal of MitoProt led to a modest decrease in performance relative to the default equal-weight scheme. These results indicate that although MitoProt contributes FPs, its inclusion provides a complementary signal that supports overall predictive performance. The modest effect of weighting adjustments further suggests that CoMR performance is not dominated by any single evidence layer. Moreover, the scoring framework is implemented as a customizable scorecard, allowing users to adapt component weights and fine-tune CoMR to maximize performance according to the characteristics of their own datasets.

**Table 1:** AUC scores of ROC curves summarizing CoMR performance on *S. cerevisiae* and *P. pyriformis* proteomes, according to different scoring schemes.

Scoring scheme	*S. cerevisiae*		*P. pyriformis*	
	ROC-AUC	ΔROC-AUC versus equal scoring	ROC-AUC	ΔROC-AUC versus equal scoring
Equal scoring	0.91782	–	0.86113	–
No MitoProt scoring	0.91482	−0.00300	0.85875	−0.00238
MitoProt down-weighted	0.92202	+0.00420	0.86571	+0.00458
No tree search scoring	0.91074	−0.00708	0.84789	−0.01324
No SMD scoring	0.91285	−0.00497	0.83138	−0.02975
No TargetP2 scoring	0.91736	−0.00046	0.86220	+0.00107
No MitoFates scoring	0.91613	−0.00169	0.86215	+0.00102
No NR search scoring	0.85265	−0.06517	0.82215	−0.03898

**Figure 5:**
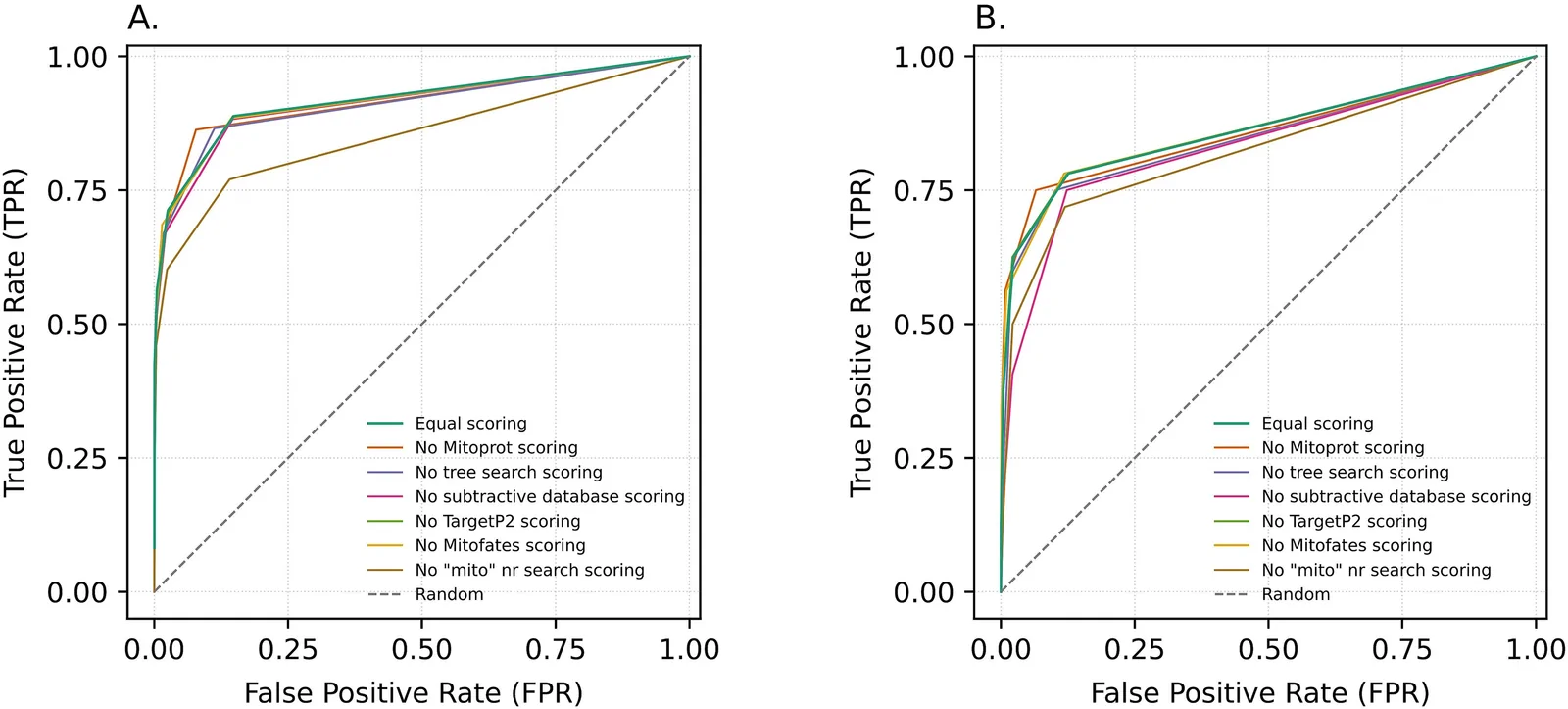
Scoring-scheme sensitivity for the *S. cerevisiae* proteome and *P. pyriformis* proteome shows that CoMR performance varies depending on component deletions; (a) *S. cerevisiae* proteome; (b) *P. pyriformis* proteome; these plots display ROC curves under the full set of scoring schemes, representing the effectiveness of the CoMR pipeline when using the equal scoring scheme, when MitoProt is removed from the scoring scheme, when the automatic phylogenetic analysis is removed from the scoring scheme, when the SMD search is removed from the scoring scheme, when TargetP2 is removed from the scoring scheme, when MitoFates is removed from the scoring scheme, and when the NCBI NR database search is removed from the scoring scheme; the dashed line indicates a random classifier.

To quantify the contribution of individual evidence layers, we systematically removed each component from the scoring scheme and recalculated ROC-AUC values for the *S. cerevisiae* proteome (Table 1 and Fig. 5a). This ablation analysis allowed us to assess the relative influence of each search strategy on overall predictive performance, with effects reported as changes in ROC-AUC relative to the default equal-weight score. Removal of NR-based homology support produced the largest decrease in ROC-AUC (ΔROC-AUC = −0.065), indicating that large-scale homology searches contribute substantially to mitochondrial protein identification in *S. cerevisiae*. Performance remained stable even under stricter genus- and order-level NR exclusion (Supplementary Fig. S1 and Supplementary Table S1), indicating that CoMR performance is not solely dependent on near-identical homologs. In contrast, removal of individual targeting predictors resulted in only minor changes in performance (|ΔROC-AUC| ≤ 0.003), while removal of the remaining evidence layers produced more moderate reductions. These results suggest that CoMR integrates complementary and partially overlapping signals rather than being dominated by any single evidence layer. This effect likely reflects the dense phylogenetic representation of fungal sequences in NR, which enhances the homology-based signal for *S. cerevisiae* proteins.

To address possible redundancy between evidence classes, we quantified the overlap between MTS-based support and homology-based support among reference mitochondrial/MRO proteins (Table 2 and Supplementary Tables S2 and S3). In *S. cerevisiae*, 408 of 744 reference mitochondrial proteins were supported by both MTS and homology evidence, while 166 were supported by homology alone and 70 by MTS predictors alone. These results indicate that targeting- and homology-based signals are partly overlapping but not redundant. This is supported by pairwise Jaccard analyses, with the strongest overlap observed between the targeting predictors TargetP2 and MitoFates (Jaccard = 0.647; Supplementary Table S4).

**Table 2:** Overlap between MTS- and homology-based support among reference mitochondrial/MRO proteins.

Dataset	MTS only (%)	Homology only (%)	MTS + homology (%)	Neither (%)	Reference positives
*S. cerevisiae*	70 (9.4)	166 (22.3)	408 (54.8)	100 (13.4)	744
*P. pyriformis*	2 (6.3)	14 (43.8)	8 (25.0)	8 (25.0)	32

The corresponding overlap analysis across all proteins is provided in Supplementary Table S2, and the full reference-positive overlap table is provided in Supplementary Table S3.

### CoMR performance on a non-model organism, *Paratrimastix pyriformis*

Because the relative contribution of individual evidence layers is likely to depend on phylogenetic context and database representation, we next evaluated CoMR performance on a divergent, non-model organism. We applied the same benchmarking framework to the anaerobic protist *P. pyriformis*, whose spatial proteomics-derived MRO proteome includes 32 annotated proteins [39].

Using the default equal-weight scoring scheme, we constructed a ROC curve and calculated a ROC-AUC of 0.86 for the *P. pyriformis* proteome (Fig. 2b and Table 1), indicating strong discriminatory performance despite the reduced size and divergence of its MRO proteome. As observed for *S. cerevisiae*, MitoProt generated an elevated number of FPs in the *P. pyriformis dataset* (Fig. 3b). Down-weighting MitoProt resulted in a modest increase in ROC-AUC (Table 1 and Fig. 4b), indicating that its contribution inflates FPRs in this proteome. In contrast to *S. cerevisiae*, where NR-based homology had the largest impact on performance by far, removal of both NR and SMD homology support produced the strongest decreases in *P. pyriformis* (ΔROC-AUC = −0.039 and −0.030, respectively; Table 1 and Fig. 5b). This pattern is consistent with a stronger dependence on complementary homology sources in this divergent lineage.

In *P. pyriformis*, complementarity was pronounced: 14 of 32 reference MRO proteins were supported by homology alone, compared with 8 supported by both evidence classes and 2 by MTS predictors alone (Table 2 and Supplementary Tables S2 and S3). Consistently, pairwise Jaccard overlaps were substantially lower overall, with a maximum overlap of 0.171 between MitoProt and MitoFates (Supplementary Table S4). These results highlight the value of integrating homology-based evidence when reconstructing MRO proteomes in divergent non-model eukaryotes.

### Performance comparison between CoMR and TargetP2

**Figure 6:**
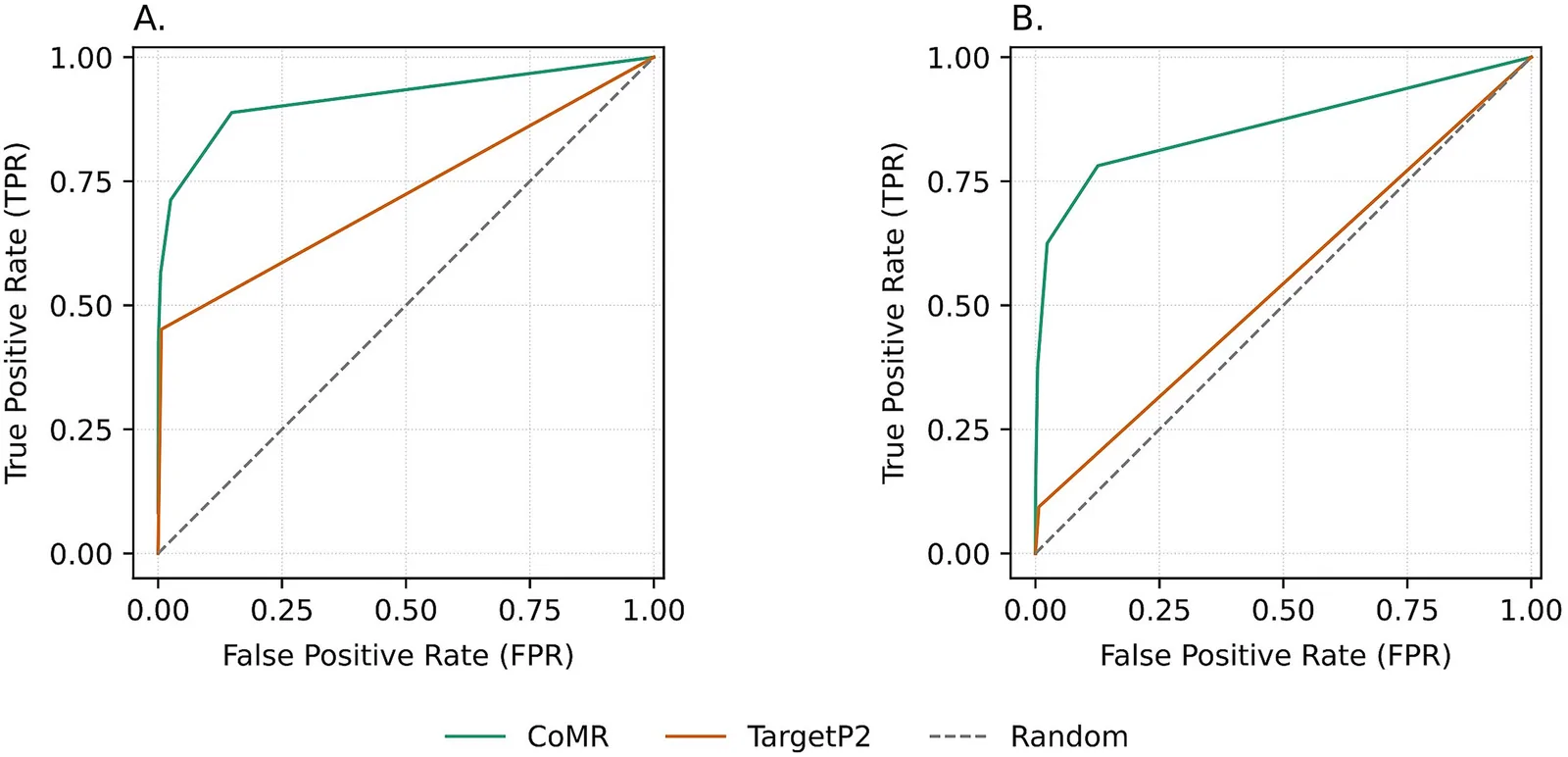
A comparison of CoMR function versus TargetP2 using the *S. cerevisiae* and *P. pyriformis* proteomes; (a) *S. cerevisiae* proteome; (b) *P. pyriformis* proteome; ROC curves for CoMR using the equal scoring scheme, TargetP2, and a random classifier are displayed.

Among the three targeting predictors included in CoMR, TargetP2 showed the lowest FPR and overall strongest standalone performance. We therefore used TargetP2 as a baseline comparator to evaluate CoMR performance on both the *S. cerevisiae* and *P. pyriformis* proteomes. For both datasets, CoMR achieved higher ROC-AUC values than TargetP2 under the equal-weight scoring scheme (Table 3 and Fig. 6a and b). This indicates that integration of multiple evidence layers provides improved discriminatory performance relative to targeting prediction alone.

**Table 3:** Comparing the performance of CoMR and TargetP2 on the *S. cerevisiae* and *P. pyriformis* proteomes using AUC-ROC scores.

ROC curves	*S. cerevisiae* AUC score	*P. pyriformis* AUC score
CoMR equal score	0.91782	0.86113
TargetP2	0.72255	0.54350

**Figure 7:**
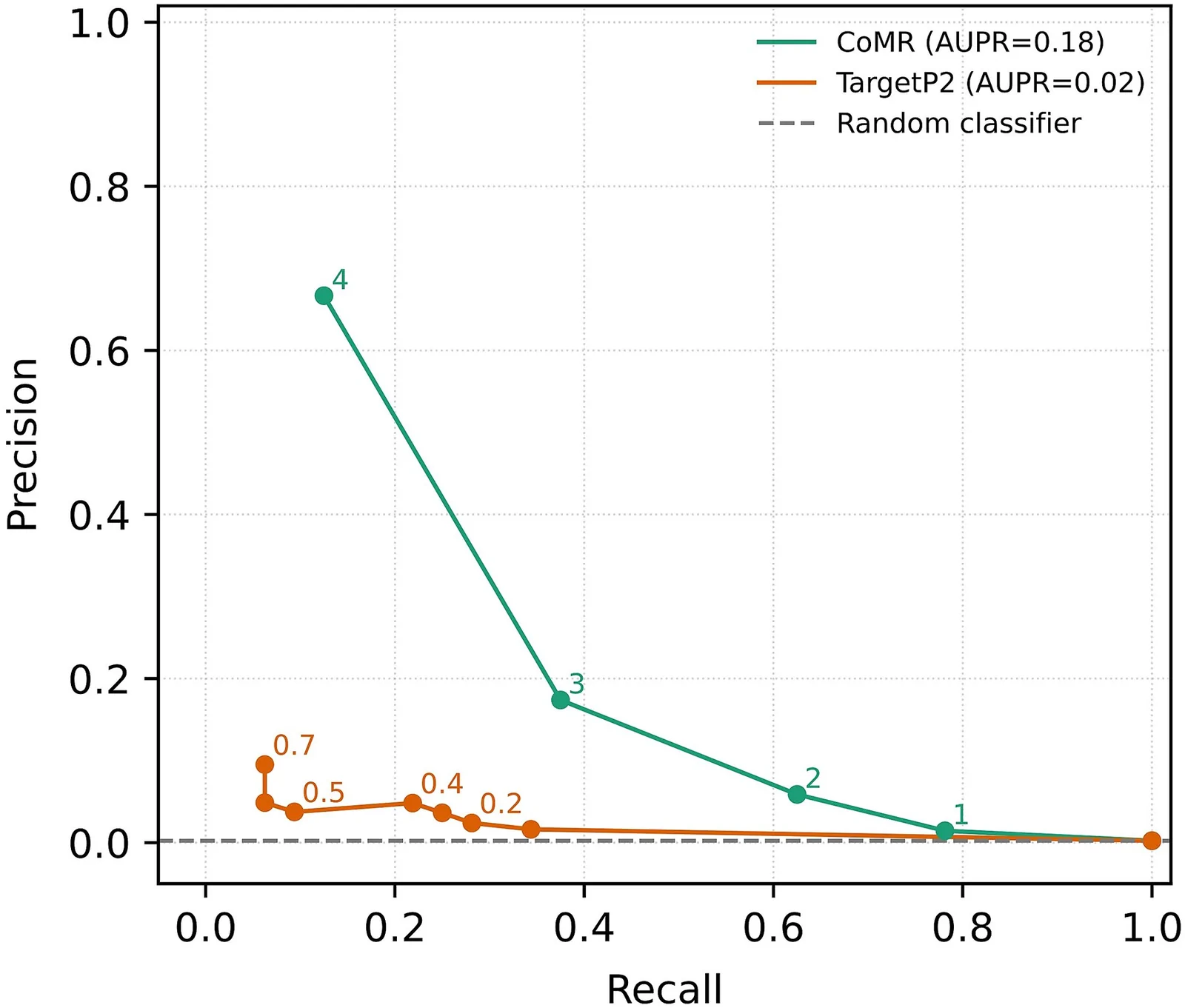
PR performance for CoMR and TargetP2 in the *P. pyriformis* proteome; PR curve for CoMR (equal scoring) and the TargetP2 PR curve (score threshold); for visualization, only thresholds yielding informative precision (i.e. non-zero, at least one TP) are plotted; the dashed line indicates a random classifier at prevalence.

Because the *P. pyriformis* dataset contains a small number of positive cases relative to the total proteome size, ROC-AUC values may overestimate predictive performance under extreme class imbalance [40]. To more directly evaluate classification precision, we calculated precision and recall across score thresholds and constructed PR curves for both CoMR (equal-weight scoring scheme) and TargetP2 (Table 4 and Fig. 7). CoMR achieved a PR-AUC of 0.18340, substantially exceeding both the random baseline (0.00236, corresponding to the prevalence of MRO proteins in the dataset) and TargetP2 (PR-AUC = 0.01859). This represents an ~78-fold enrichment over random expectation and nearly a 10-fold improvement over standalone targeting prediction. These results indicate that CoMR maintains strong PR performance despite the extreme rarity of MRO proteins in the *P. pyriformis* proteome.

**Table 4:** Area under the PR curve scores comparing the performance of CoMR and TargetP2 on the *P. pyriformis* proteome.

PR curve	AUC score
*P. pyriformis* CoMR	0.18340
*P. pyriformis* TargetP2	0.01859
Random classifier (TP/all data)	0.00236

### Top-ranked CoMR candidates are enriched for mitochondrial proteins

To assess CoMR as a candidate-prioritization tool, we evaluated the enrichment of reference mitochondrial/MRO proteins among top-ranked candidates (Table 5 and Supplementary Table S5). In *P. pyriformis*, 8 of the top 10 candidates were experimentally supported MRO proteins, corresponding to 80% precision and a 338-fold enrichment over random expectation. The top 100 candidates recovered 20 of 32 reference MRO proteins, and the top 500 recovered 25 of 32 proteins. In *S. cerevisiae*, CoMR recovered 99 curated mitochondrial proteins among the top 100 candidates and 474 among the top 500, while the top 1000 recovered 661 of 744 reference mitochondrial proteins. These results show that CoMR enriches true mitochondrial/MRO proteins among high-ranking candidates and supports practical prioritization for expert curation.

**Table 5:** Enrichment of reference mitochondrial/MRO proteins among top-ranked CoMR candidates.

Dataset	Top N	TPs	Precision (%)	Recall (%)	Fold enrichment
*S. cerevisiae*	100	99/100	99.0	13.3	7.9×
*S. cerevisiae*	500	474/500	94.8	63.7	7.6×
*S. cerevisiae*	1000	661/1000	66.1	88.8	5.3×
*P. pyriformis*	10	8/10	80.0	25.0	338.3×
*P. pyriformis*	100	20/100	20.0	62.5	84.6×
*P. pyriformis*	500	25/500	5.0	78.1	21.1×

Selected top-*N* thresholds are shown to highlight early precision and broader recovery of reference positives; the complete top-*N* analysis is provided in Supplementary Table S5.

### CoMR runtime

To assess the computational cost of large-scale homology searches, we evaluated CoMR runtime and memory usage under different resource allocations and with or without NR searches enabled (Table 6). Under high-performance settings (128 central processing units (CPUs); DIAMOND threads = 64; block size = 4), complete execution required 3 h 12 min for *S. cerevisiae* (5967 proteins) and 5 h 11 min for *P. pyriformis* (13 532 proteins), with peak memory usage of 200 and 228 GB, respectively. Reducing computational allocation (24 CPUs; DIAMOND threads = 8; block size = 1) increased runtime and reduced memory consumption. For *S. cerevisiae*, runtime increased from 3 h 12 min to 7 h 26 min, while peak RAM decreased from 200 to 88 GB. A similar scaling trend was observed for *P. pyriformis*, with runtime increasing from 5 h 11 min to 10 h 27 min and peak RAM decreasing from 228 to 115 GB.

**Table 6:** Computational performance of CoMR across hardware configurations.

Proteome	#Proteins	NR	DIAMOND threads	DIAMOND block size	#CPU	Total runtime (hh:mm)	Peak RAM (GB)
*S. cerevisiae*	5967	Yes	64	4	128	03:12	200
*S. cerevisiae*	5967	Yes	8	1	24	07:26	88
*S. cerevisiae*	5967	No	8	1	24	04:10	81
*P. pyriformis*	13 532	Yes	64	4	128	05:11	228
*P. pyriformis*	13 532	Yes	8	1	24	10:27	115
*P. pyriformis*	13 532	No	8	1	24	09:40	109

CoMR was executed on the *S. cerevisiae* (5967 proteins) and *P. pyriformis* (13 532 proteins) proteomes under two hardware configurations: a high-performance computing cluster and under a reduced-performance allocation. The table reports the number of DIAMOND threads and block size used for the NR homology search, the total number of CPU cores allocated, total runtime, and peak memory usage (GB). Runtime corresponds to the complete pipeline execution including targeting prediction, homology searches, phylogenetic reconstruction, and score integration.

Disabling NR searches under reduced-resource settings moderately reduced runtime and memory usage. In *S. cerevisiae*, runtime decreased from 7 h 26 min to 4 h 10 min when NR was disabled, while peak RAM decreased from 88 to 81 GB. In *P. pyriformis*, runtime decreased from 10 h 27 min to 9 h 40 min and peak RAM decreased from 115 to 109 GB. Although NR searches represent a major computational component, substantial runtime persisted even when NR was disabled, particularly for the larger *P. pyriformis* proteome. This likely reflects the computational cost of multiple sequence alignment and phylogenetic tree reconstruction steps, which scale with the number of candidate protein families identified.

Based on these benchmarks, full CoMR runs including NR searches are best suited to high-performance computing (HPC) environments, with the reduced-resource configuration tested here providing a practical reference point for proteomes of ~5000–15 000 proteins. For exploratory analyses or when NR searches are computationally limiting, users can disable NR searches (enable_nr = false) and run CoMR using targeting predictions, SMD searches, HMM profiles, and phylogenetic evidence. Detailed recommendations for tuning CPU allocation, DIAMOND threads, block size, and workflow-specific settings across workstation and HPC environments are provided in the CoMR GitHub documentation (https://github.com/theLabUpstairs/CoMR).

## Discussion

Mitochondrial proteome reconstruction remains challenging, particularly in phylogenetically diverse or poorly represented eukaryotic lineages. Here, we introduce CoMR, an integrative workflow that combines targeting prediction, curated homology searches, large-scale NR searches, and automated phylogenetic analysis into a unified scoring framework. Across both a well-annotated model organism (*S. cerevisiae*) and a divergent anaerobic protist (*P. pyriformis*), CoMR demonstrated strong discriminatory performance and consistent improvement over standalone targeting prediction.

### Integrative scoring improves over targeting prediction alone

In *S. cerevisiae*, CoMR achieved an ROC-AUC of 0.92, substantially outperforming TargetP2 (ROC-AUC = 0.72). This difference underscores a central finding of this study: MTSs alone are insufficient for comprehensive proteome reconstruction. While targeting predictors performed well in minimizing FNs, their FPRs varied considerably, particularly for MitoProt. The modest changes observed when altering MitoProt weighting demonstrate that CoMR is robust to individual predictor biases, likely because independent evidence layers buffer one another. This integrative behavior is a key advantage over single-method approaches.

### Evidence-layer contributions are lineage dependent

Ablation analyses revealed that the relative importance of evidence layers varies across phylogenetic contexts. In *S. cerevisiae*, removal of NR-based homology had the largest impact on ROC-AUC, illustrating the dense representation of fungal sequences in public databases. In contrast, in *P. pyriformis*, both NR and curated SMD homology contributed substantially to predictive performance. This shift likely reflects reduced representation of metamonad sequences in NR and highlights the value of curated mitochondrial reference datasets in poorly sampled lineages. These results emphasize that no single evidence type is universally dominant. Instead, optimal mitochondrial prediction depends on phylogenetic context and database coverage. The ability of CoMR to integrate multiple partially independent signals appears particularly beneficial in non-model organisms; however, CoMR will be influenced by the composition of public sequence databases, particularly NR. Although taxonomic exclusion reduces circularity during benchmarking, it cannot remove broader biases caused by uneven taxonomic sampling. NR-derived hits should therefore be interpreted as one evidence layer within CoMR rather than as definitive support for mitochondrial localization, especially when applying the workflow to lineages with limited representation in public databases.

### Performance under extreme class imbalance

The *P. pyriformis* dataset presents a highly imbalanced classification problem, with only 32 annotated MRO proteins among ˃13 000 sequences. While ROC-AUC values remained strong (0.86), PR analysis provided a more informative assessment. CoMR achieved a PR-AUC of 0.183, representing a ~78-fold enrichment over random expectation and nearly a 10-fold improvement over TargetP2. These results demonstrate that CoMR maintains meaningful precision even when TPs are rare, an essential property for mitochondrial reconstruction in reduced or highly derived organelles.

### Robustness and customizability of the scoring framework

Down-weighting or removing individual evidence layers resulted in only modest changes in performance, suggesting that CoMR is not overly dependent on any single signal. This robustness supports the use of the default equal-weight scoring scheme across diverse datasets. At the same time, the customizable scorecard architecture allows users to adjust component weights to reflect lineage-specific knowledge or data availability. Such flexibility may be particularly valuable when investigating deeply branching or sparsely sampled eukaryotic groups.

### Limitations

Several limitations should be acknowledged. First, benchmarking relied on curated proteomes, which may themselves be incomplete or subject to annotation bias. Second, NR-based evidence is inherently influenced by database composition; although taxonomic exclusion was implemented to prevent circularity, residual biases toward well-represented lineages remain unavoidable. Third, the automated phylogenetic component should be interpreted as supporting evidence for candidate prioritization rather than definitive validation. Candidate placement may be sensitive to alignment quality, taxon sampling, trimming, model choice, and tree uncertainty, particularly for divergent or poorly aligned proteins. Although CoMR retains unresolved mitochondrial/other ambiguity as undefined, it does not explicitly model topology or branch-support uncertainty. High-priority or unexpected candidates should therefore be inspected manually.

Future improvements could incorporate additional lineage-specific reference datasets. Experimental validation in additional non-model systems would further strengthen generalizability.

## Conclusion

Together, these results demonstrate that integrative evidence scoring substantially improves mitochondrial proteome reconstruction over standalone targeting prediction, particularly in non-model and database-sparse lineages. CoMR provides a reproducible and flexible framework for mitochondrial and MRO prediction, enabling systematic comparative analyses across diverse eukaryotes.

**Key Points**

- Comprehensive Mitochondrial Reconstructor (CoMR) is an integrative scoring pipeline that combines targeting prediction, curated and large-scale homology searches, and automated phylogenetic analysis for mitochondrial proteome reconstruction.
- In *S. cerevisiae*, CoMR achieved strong discriminatory performance [receiver operating characteristic (ROC)-area under the curve (AUC) = 0.92], outperforming standalone prediction with TargetP2, a predictor of N-terminal targeting peptides (ROC-AUC = 0.72).
- In the divergent metamonad *Paratrimastix pyriformis*, CoMR maintained robust performance (ROC-AUC = 0.86; precision-recall-AUC = 0.183) despite extreme class imbalance.
- Ablation analyses demonstrate that the relative contribution of evidence layers is lineage-dependent, highlighting the importance of integrative approaches in poorly represented clades.
- CoMR provides a reproducible and scalable framework applicable to both model and non-model eukaryotes.

## Supplementary Material

CoMR_Supplementary_Material_BiB_final_bbag474

## Data Availability

The CoMR workflow, including all scripts, configuration templates, and Snakemake rules, is publicly available via the CoMR GitHub repository (https://github.com/theLabUpstairs/CoMR). CoMR databases and benchmarking-modified databases and associated scripts are publicly available via the CoMR Figshare repository (https://doi.org/10.17044/scilifelab.31361839). All software dependencies are containerized, and reproducible execution is supported via Docker (ghcr.io/thelabupstairs/comr:git-2bc33bf) or Singularity/Apptainer image, available from the CoMR Figshare repository (https://doi.org/10.17044/scilifelab.31361839). Documentation, installation instructions, and usage examples are provided within the CoMR github repository, including system-specific execution guides and workflow descriptions. These resources are intended to support reproducible deployment and adaptation of the pipeline to diverse computational environments.
